# Fungi in Permafrost-Affected Soils of the Canadian Arctic: Horizon- and Site-Specific Keystone Taxa Revealed by Co-Occurrence Network

**DOI:** 10.3390/microorganisms9091943

**Published:** 2021-09-13

**Authors:** Milan Varsadiya, Tim Urich, Gustaf Hugelius, Jiří Bárta

**Affiliations:** 1 Department of Ecosystems Biology, University of South Bohemia, 37005 České Budějovice, Czech Republic; mvarsadiya@prf.jcu.cz; 2 Institute of Microbiology, University of Greifswald, 17487 Greifswald, Germany; tim.urich@uni-greifswald.de; 3 Department of Physical Geography, Stockholm University, 10691 Stockholm, Sweden; gustaf.hugelius@natgeo.su.se; 4 Bolin Centre for Climate Research, Stockholm University, 10691 Stockholm, Sweden; 5 Centre for Polar Ecology, Faculty of Science, University of South Bohemia, 37005 České Budějovice, Czech Republic

**Keywords:** arctic, permafrost, keystone taxa, co-occurrence network, Zi-Pi plot

## Abstract

Permafrost-affected soil stores a significant amount of organic carbon. Identifying the biological constraints of soil organic matter transformation, e.g., the interaction of major soil microbial soil organic matter decomposers, is crucial for predicting carbon vulnerability in permafrost-affected soil. Fungi are important players in the decomposition of soil organic matter and often interact in various mutualistic relationships during this process. We investigated four different soil horizon types (including specific horizons of cryoturbated soil organic matter (cryoOM)) across different types of permafrost-affected soil in the Western Canadian Arctic, determined the composition of fungal communities by sequencing (Illumina MPS) the fungal internal transcribed spacer region, assigned fungal lifestyles, and by determining the co-occurrence of fungal network properties, identified the topological role of keystone fungal taxa. Compositional analysis revealed a significantly higher relative proportion of the litter saprotroph *Lachnum* and root-associated saprotroph *Phialocephala* in the topsoil and the ectomycorrhizal close-contact exploring *Russula* in cryoOM, whereas Sites 1 and 2 had a significantly higher mean proportion of plant pathogens and lichenized trophic modes. Co-occurrence network analysis revealed the lowest modularity and average path length, and highest clustering coefficient in cryoOM, which suggested a lower network resistance to environmental perturbation. Zi-Pi plot analysis suggested that some keystone taxa changed their role from generalist to specialist, depending on the specific horizon concerned, *Cladophialophora* in topsoil, saprotrophic *Mortierella* in cryoOM, and *Penicillium* in subsoil were classified as generalists for the respective horizons but specialists elsewhere. The litter saprotrophic taxon *Cadophora finlandica* played a role as a generalist in Site 1 and specialist in the rest of the sites. Overall, these results suggested that fungal communities within cryoOM were more susceptible to environmental change and some taxa may shift their role, which may lead to changes in carbon storage in permafrost-affected soil.

## 1. Introduction

Fungi are ubiquitous and one of the most species-rich groups of organisms in the Arctic soil ecosystem [1]. Our knowledge of their role in soil organic matter transformation is continually increasing, still, there are many unanswered questions regarding the relationship between different taxa with distinct lifestyles (i.e., saprotrophs, mycorrhizae) as they are thought to be the key players of elemental and energy flow in carbon (C) and nitrogen (N) cycles. They also influence the occurrence of other microbes, such as bacterial decomposers, pathogens, and symbiotrophs [2,3,4]. Despite the ubiquitous distribution of fungi in the soil, our knowledge of their biodiversity and functional traits in permafrost-affected soil (PAS) remains limited to relatively few studies. Nevertheless, the number of fungal studies from PAS is continually increasing, including studies from Svalbard [5,6], Alaska [7,8], and Greenland [9,10]. However, only a few specific studies, from Eastern Siberia [11] and the Northern American Arctic transect [12], have studied the fungal community from the buried organic matter (cryoOM) pocket, which stores a significant amount (approx. 470 Pg C) of organic C due to cryoturbation of the top organic layer.

Most ecological studies have focused on functional diversity, as opposed to biodiversity only, due to the fact that individual species can have several functions in an ecosystem [13,14]. It is well-known that many fungal species play redundant roles by altering or manipulating the distribution of the same soil resource [15]. Several sequence-based studies have parsed operational taxonomic units (OTUs) into more ecologically meaningful groups [16,17,18]. These groups would have a similar function in the ecosystem and can be divided into symbiotrophs, pathotrophs, and saprotrophs, collectively called trophic modes [19]. These trophic modes of fungi play critical roles in the Arctic, for example, symbiotrophs help plants to uptake nutrients, especially N, which is considered growth-limiting in the Arctic tundra [20]. On the other hand, saprotrophic fungi are essential for decomposing dead plant biomass and, therefore, crucial for nutrient and carbon cycling in the Arctic soil [21,22,23]. Pathotrophic fungi are known to infect other fungi to gain organic carbon and by doing so, they control other trophic modes [24]. There is still a lack of data on the occurrence and potential interactions of these trophic guilds in Arctic PAS.

Microbes in the soil create a complex ecological network by interacting with each other [25]. This interaction includes predation, competition, parasitism, and mutualism [26,27,28,29,30]. To predict the composition of ecological microbial networks, it is especially important to understand the microbial assembly, the potential interactions of keystone taxa, and the resulting ecological function [29,31,32]. Despite the importance of these interactions in ecological functions, the direct detection and investigation of these interactions are difficult [25,33]. Several studies have demonstrated that the specific properties of ecological species networks can, at least to some extent, explain the real response of the microbial community to environmental changes [34,35,36]. For example, a study of experimental warming from Alaskan tundra soil evidenced that warming conditions had a more complex and denser bacterial co-occurrence network compared to the control site, while the opposite was observed for the fungal network [36]. The authors suggested that the environmental changes were associated with a distinct response by microbial communities [36].

The specific properties (topological properties) of the co-occurrence networks include (1) the degree distribution, which determines how many other taxa in the network are connected with the given taxa; (2) the clustering coefficient, which describes how well a taxon is connected to its neighboring taxa (analogy to human society, the clustering coefficient is a measure of an “all-my-friends-know-each-other” property); (3) the average path length, which is the shortest path between the two most distant taxa in the network (a short average path length facilitates the quick transfer of information and reduces costs leading to the concept of a small world where everyone is connected to everyone else through a very short path); (4) modularity, which measures the degree to which the network was organized into clearly delimited modules. Networks with high modularity have dense connections between the taxa within modules but sparse connections between taxa in different modules [37].

Another aspect of the ecological network is the identification of keystone species, connectors and modular hubs [38,39]. The connectors are defined as those taxa or nodes which have more connections among different modules, in contrast, module hubs are those taxa or nodes which have more connections within their own modules [27]. These keystone taxa play a key role in modulating network structure and function, as they often have dominant relationships and interactions among other taxa [27]. The network analysis may also provide information about the importance of low abundant taxa for supporting the structure and functions of microbial communities. Most soil ecosystem studies have concentrated on the most abundant microbial species [11,40,41,42]. However, low abundance taxa play a significant role in maintaining ecosystem functions, despite their low proportion. Therefore, some of them are also considered as keystone taxa [31,43]. Herren and co-authors [44] suggested that keystone taxa can explain microbiome compositional turnover better than the most abundant taxa combined. The keystone species are most important to protect since their absence might lead to network fragmentation [45]. For instance, the disappearance of a keystone species from a network of bacterial wilt-susceptible soil made it more loose and unstable compared to a network of healthy soil that had more keystone species [46].

To understand the complexity of these interactions in fungal communities in Arctic PAS, we addressed the following questions: (1) Does each horizon type (topsoil, cryoOM, subsoil, and permafrost) contain exclusive/unique fungal genera and lifestyles? (2) Do network topological properties significantly differ between different horizons and tundra sites? (3) Which are the keystone species in different horizons and tundra sites? (4) Is there any correlation between network modules, keystone species, and environmental factors?

To address these questions, we collected 122 soil samples from four different horizons of four distinct tundra sites from Herschel Island, Canada. We used Illumina MiSeq sequencing data of the fungal ribosomal internal transcribed spacer (ITS) to analyze the change in the fungal community composition and intertaxa interaction. We implemented sequencing data to infer fungal community composition, functional guild distribution, and microbial ecological network analysis. Our central objective was to characterize and understand the microbial ecological network pattern of sequencing data obtained from Illumina MiSeq sequencing and specific emphasis was given to cryoOM.

## 2. Materials and Methods

### 2.1. The Site Description and Soil Sampling

The study area is located on Herschel Island (Qikiqtaruk; 69°34′ N, 138°55′ W, Beaufort Sea, Canada). The mean annual air temperature is −9 °C with the mean monthly air temperature varying between −26.3 °C (February) and 8.7 °C (July). The mean annual precipitation ranges between 150 and 200 mm [47].

During late summer, a total of 122 samples were collected from four tundra sites and three different types of soil horizons of the active layer. These horizons represented upper topsoil, cryoOM, and mineral subsoil based on field description. We also collected samples from the permafrost. The four sites had a landscape of hummocky tussock tundra (Site 1), slightly disturbed upland tundra dominated by non-sorted circles (Site 2), wet polygonal tundra (Site 3), and hummocky tussock tundra dominated by nonsorted circles (Site 4). The main vegetation types were from Site 1, moss and cotton grass; Site 2, Arctic willow and *Dryas-Vetch*; Site 3, Carex and bryophytes as primary vegetation types; and Site 4, *Ledum palustre* and *Betula nana*. The different landscape types and the variability of soil properties in the landscape are described in detail by Siewert et al. [48].

Soil samples were collected from four horizons of permafrost-affected soil which included topsoil; cryoOM; subsoil; and permafrost. We collected samples according to protocol described by Schoeneberger et al. [49] and we employed additional methods to acquire soil samples from permafrost [50,51]. A detailed description of the sampling location and sampling protocol was described in our previous study [52].

### 2.2. Measurement of Environmental Factors

We dried and reweighed soil samples at 60 °C for 48 h to determine the moisture content. Soil pH was measured by pH meter 3151i (Xylem incorporation GmbH, Hessen, Made in Germany) in soil suspension with a ratio of 1:2.5 (*w*/*v*). Total carbon (Ctot) and nitrogen (Ntot) content were determined from 60 °C dried soil sample (8–10 mg) using an Elementar Vario Micro cube (Elementar, Langenselbold, Germany) and expressed in percentage. The carbon to nitrogen ratio (C/N ratio) was calculated by dividing Ctot with Ntot. The dissolved organic carbon (DOC) and dissolved nitrogen (DN) were analyzed by mixing soil: water in a 1:5 ratio (*w*/*v*) and shaking on an orbital shaker (150 rpm) for an hour and the filtered soil solution (10–15 mL) was used for LiquiTOCII (Elementar, Germany) and expressed in ug g^−1^ dry weight of soil.

### 2.3. Extracellular Enzymes Activities

Hydrolytic enzymes involved in degradation of organic molecules like cellulose, chitin, protein, and lignin were measured by microplate fluorometric assays according to Barta et al. [53]. We used a half gram of sieved soil suspended in 50 mL of distilled deionized nuclease-free water (ddH_2_O) and ultrasonicated at low energy (120 W) for 4 min. Potential activities of β-glucosidase (BG), 1, 4-β-cellobiohydrolase (CBH), chitinase (NAG), and leucine aminopeptidase (LAP) were measured fluorometrically using 4-methylumbelliferyl- (MUF) and aminomethylcoumarin (AMC) as substrates (50–300 uM), respectively [54]. A 200 μL sample of the soil suspension and 50μL substrate (β-D-glucopyranoside, N-cellobiopyranoside, phosphate, N-acetglucosaminide, and L-leucine-7-amido-4-methyl coumarin, respectively) were pipetted into black microtiter plates in 3 analytical replicates. For each sample, a standard curve with methyl umbelliferyl was used for the calibration of ß-glucosidase, cellobiohydrolase, chitinase, whereas aminomethylcoumarin was used for the calibration of leucine amino-peptidase. Plates were incubated in the dark for 30 min and the first fluorescence was measured at 465 nm emission at an excitation of 360 nm (Tecan Infinite F200 fluorimeter, Schoeller instruments, Prague-Kunratice, Czech Republic). Fluorescence was measured again after 60 and 120 min. Enzyme activities were measured nmol g^−1^ dry weight of soil h^−1^.

### 2.4. DNA Extraction and Quantitative Assessment of Fungal Community by qPCR

We extracted total genomic DNA from all collected soil samples (appx. 0.25 g) using a DNeasy PowerSoilTM DNA Isolation Kit (Qiagen, Düsseldorf, Germany). Extracted DNA was stored at −20 °C for further use. The 18S rDNA was used to amplify total fungal abundance in the sample, each reaction was performed with 20 μL of reaction mixture containing 3 μL of DNA from soil samples. The fungal ribosomal gene was amplified using a nu-SSU-0817-5′/nu-SSU-1196-3′ primer set [55]. We used melt curve and gel electrophoresis analysis to confirm the product specificity and amplicon size, respectively. Standards were made from 10-fold dilution of a known amount of purified PCR product obtained from *Aspergillus niger*. The qPCR assay was performed in two replicates for each sample, along with standard and control (non-template ddH_2_Owater).

### 2.5. Barcoded Amplicon Sequencing

Aliquots of DNA extracts were sent to the SEQme Company (Dobříš, Czech Republic) for the preparation of a library and sequencing using the MiSeq2500 platform. The Earth Microbiome Project (EMP) protocol was used for library preparation with modified universal primers ITS1F/ITS2 [56]. The fungal ITS1 region was extracted from reads using the ITSx algorithm [57]. Amplicons were trimmed to equal lengths (150bp) and fungal unique reads were grouped to zero-radius OTUS (zOTUs) using a UNOISE 3.0 algorithm [58,59], which also included the removal of potential chimeric sequences. The taxonomic assignment of each fungal zOTUs was performed using the BLAST algorithm (E-value = 0.001) in UNITE [60]. Raw sequencing data were deposited in the European Nucleotide Archive (ENA) under the PRJEB44296 study.

Species richness (Chao1), diversity (Shannon), and evenness (Simpson) were calculated using the “microbiome” package [61] in R 3.5.3 [62]. To determine if the specific functional groups of fungi differed between different horizons and tundra sites, we classified each zOTU into trophic modes and lifestyles using the fungal functional database FungalTraits [63].

### 2.6. Network Construction

To better understand the fungal communities’ interaction across different horizons and tundra sites, we constructed the fungal ecological network by calculating all possible Spearman correlation coefficients between zOTUs. To increase the robustness of the ecological network, we used only those zOTUs that were present in more than 30% of the sample (each horizon and tundra sites), and relative proportions of less than 0.1% were also excluded from the analysis. Spearman’s Rho between the pairwise zOTUs matrices were constructed using the “Hmisc” package [64] in R. The false discovery rate (FDR) controlling procedure was used to calculate the *p*-values for multiple testing [65]. A valid co-occurrence was considered to be robust if the absolute value of the Spearman correlation coefficient was either equal or greater than 0.6 or −0.6 and statistically significant if *p*-values < 0.01. The cut-off correlation of 0.6 or −0.6 was chosen to increase the confidence for strong fungal interactions. Network images were generated in R with the help of the “igraph” package [66]. In the network, nodes represented zOTUs, whereas edges represented the correlation between nodes. We used the undirected network (where the edge has no direction) and the Fruchterman–Reingold layout. The topology properties of the co-occurrence networks, positive edge, negative edge, total node, average path length (APL), degree distribution (DD), average closeness (AC), average betweenness (AB), edge density (ED), diameter (D), clustering coefficient (CC), number of modules, and modularity (M) were calculated using the “igraph” package [66] in R. We also constructed a random network with the same node and edges from a real biological network to determine whether our biological networks were not random networks and represented the actual fungal interactions in soil. We used the “erdos. renyi. game” function from the igraph package to generate a thousand random networks and calculated APL, CC, and M.

Different nodes in the network play different topological roles. These topological roles can be described by two parameters. First is the within-module connectivity (Zi) which describes how well a node is connected with other nodes within its own module. The second parameter is connectivity between modules (Pi) which suggests how well a node is connected to different modules. The threshold values of Zi and Pi for categorizing nodes into different topological roles are 2.5 and 0.62, respectively, according to previous studies [67,68,69,70]. In general, the topological role of each node subdivides into four categories according to pollination networks [70]. These categories are: (1) peripheral nodes (specialist), which have low Zi (<2.5) and Pi values (<0.62) (i.e., they have only a few edges that are always connected to the node within their modules); (2) connectors (generalist), which have a low Zi (<2.5) but a high Pi value (>0.62) (i.e., these nodes tend to have more connections with several modules); (3) module hubs (generalist), which have a high Zi (>2.5) but a low Pi value (<0.62) (i.e., these are the nodes which have more connections with other nodes but within their own modules); (4) network hubs (supergeneralist), which have both high Zi (>2.5) and Pi (>0.62) values (i.e., they are connector and module hubs). The generalists (connectors, module hubs) and supergeneralist (network hubs) are considered the key microorganisms (keystone), which maintain network stability and play pivotal roles [71].

### 2.7. Statistical Analyses

The difference in environmental factors, fungi gene copies, and α-diversity indices were assessed using one-way ANOVA and followed by Tukey’s HSD post hoc test. A significant difference was considered at *p* < 0.05 unless indicated otherwise. However, we provide precise *p*-values wherever possible. We performed Spearman correlation of the log-transformed environmental factors with network modules (top five) and keystone taxa (identified from the Zi-Pi plot) using the “Hmisc” package [64] in R. A permutational analysis of variance (PERMANOVA) test was used to evaluate the linkage between fungal community composition and environmental factors using the Bray−Curtis dissimilarity matrix. The PERMANOVA test was performed by the “adonis” function in the R package “vegan” [72]. The best environmental factors explaining the fungal community composition were determined by the forward selection method. STAMP software was used to identify the difference in the mean proportion of genera and lifestyle between different horizons and tundra sites [73].

## 3. Results

### 3.1. Environmental Variables

In general, the soil samples from the topsoil had significantly greater moisture, DOC, Ctot, Ntot, and C/N ratio and followed the order topsoil > cryoOM > subsoil > permafrost. In contrast, the DN was significantly lower in the topsoil compared to other horizons. The soil samples from cryoOM had significantly greater moisture, Ctot, and Ntot compared to those from the surrounding mineral subsoil. In comparison to other horizons, the permafrost samples had the highest values for pH and DN (Table 1). The enzymatic activity of BG and LAP was significantly greater in the topsoil and decreased in the order of topsoil > cryoOM > subsoil > permafrost. The CBH and NAG activities were similar between topsoil and cryoOM, and both horizons had significantly greater activities of these enzymes than subsoil and permafrost (Table 1).

The individual horizon also had significant differences between each tundra site, the topsoil from Site 2 had significantly lower moisture, but significantly higher pH, BG, CBH, and LAP (Table S1). For cryoOM, the only significant difference between different tundra sites was found for pH and C/N ratio, Site 2 significantly had the highest pH value whereas Site 4 had, significantly, the highest C/N ratio. The lower mineral subsoil had a significant difference between the tundra sites for pH, DN, C/N ratio, CBH, and NAG.

### 3.2. Fungal Gene Abundance, Community Composition, and Diversity Differed between Horizons and Sites

**Figure 1:**
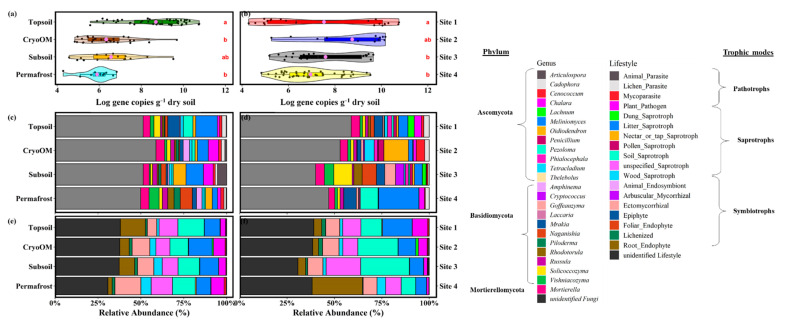
Fungi gene abundance and community composition. Log-transformed fungal gene copies per gram dry weight of soil are shown for (**a**) horizon and (**b**) tundra site. Based on Tukey’s HSD post hoc tests, gene abundance that differed between horizons and tundra sites was represented by different letters. The lavender point inside the bar plot suggested a mean value. The relative fungal taxonomic composition at genus level and fungal lifestyle for (**c**,**e**) horizon and (**d**,**f**) tundra sites were shown, respectively. Only those genera which had >1% relative proportion and filled to 100% were shown, whereas all fungal lifestyles were depicted.

Fungal 18S rRNA gene abundance was determined by quantitative PCR (qPCR), in total 104 samples were successfully amplified from 122 soil samples (Figure 1a,b). Average fungal SSU gene copies per gram of dry soil per individual soil horizon decreased in order: topsoil (5.7 ± 11.5 × 10^9^) > subsoil (2.2 ± 8.3 × 10^8^) > cryoOM (1.9 ± 9.2 × 10^8^) > permafrost (1.7 ± 2.0 × 10^6^), whereas Site 1 had a significantly higher fungal gene abundance (9.1 ± 8.2 × 10^9^) compared to the other sites.

The complete data set of fungal composition contained 858,309 filtered sequences, in which 3199 zero radius OTU (zOTUs) were affiliated to 11 fungal phyla (Table S2). Those phyla which had at least 1% of relative proportion were: Ascomycota, Basidiomycota, Mortierellomycota, and Rozellomycota.

In total, we identified 366 genera, 24 of which had more than 1% relative proportion (Figure 1c,d, Table S2). The most dominant genera belonged to the phyla Ascomycota, Basidiomycota, and Mortierellomycota. The root-associated genus *Lachnum* and endophytic fungus genus *Phialocephala* had a significantly greater mean proportion in topsoil, whereas the ectomycorrhizal genus *Russula* had a greater mean proportion in cryoOM compared to all other horizons (Welch’s *t*-test, two-sided, *p* < 0.05, Figure S1). Individual tundra sites also deferred significantly at genera levels (Figure 1d). For example, the genus that had the greatest mean proportion included ectomycorrhizal genus *Amphinema* from Site 1, soil saprotrophic genus *Oidiodendron* from Site 2, unspecified saprotrophic genus *Rhodotorula* from Site 3, and root endophytic genus *Meliniomyces* from Site 4 (Welch’s *t*-test, two-sided, *p* < 0.05, Figure S1).

Using the fungal functional database FungalTraits, we were able to assign those zOTUs that were classified into genera to trophic modes (i.e., pathotrophs, saprotrophs, and symbiotrophs) (Figure 1e,f, and Table S2). In total, we were able to assign 56.6% of zOTUs to trophic modes. Of these, roughly one-third of the assigned zOTUs, pathotrophic, saprotrophic, and symbiotrophic fungi accounted for approximately 8.4%, 29.7%, and 18.4%, respectively, on average. The pathotrophs were mainly dominated by the plant pathogens and their proportion was significantly lower in topsoil (Welch’s *t*-test, two-sided, *p* < 0.05, Figure S2). The root endophytes had a greater mean proportion in topsoil compared to other horizons, however, this difference was nonsignificant. We found a significantly greater mean proportion of ectomycorrhizal and wood saprotrophs in cryoOM compared to topsoil (data not shown). We did not find any significant difference in fungal trophic modes between cryoOM and subsoil. The relative proportion of plant-pathogen and litter saprotrophs decreased from Site 1 to Site 4, whereas the relative proportion of soil saprotrophs increased from Site 1 to Site 3 (Figure 1f). We found a significantly greater mean proportion of litter saprotrophs, plant pathogen, and lichenized trophic modes in Site 1 compared to all other sites (Welch’s *t*-test, two-sided, *p* < 0.05, Figure S2). On the other hand, Site 2 and Site 3 had a significantly greater mean proportion of plant-pathogen and lichenized and soil saprotrophs, respectively.

The alpha diversity index suggested that fungal communities from topsoil were more rich (nonsignificant chao1 index) but significantly less evenly (Simpson evenness index) distributed compared to other horizons and the opposite was true for cryoOM (Table S3). Tundra sites also significantly differed for alpha diversity indices, Site 1 had significantly higher richness and diversity whereas Site 4 had the lowest.

**Figure 2:**
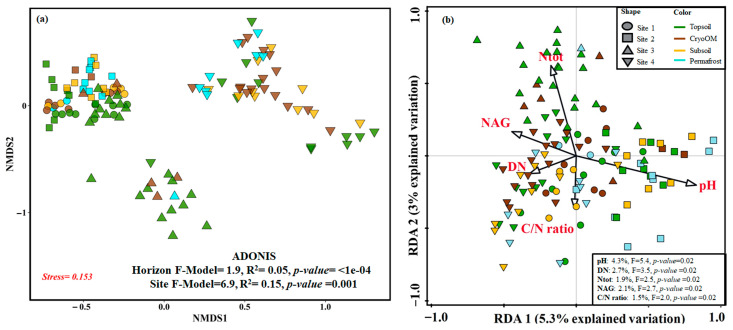
The phylogenetic dissimilarity between soil horizons and tundra types. (**a**) NMDS of fungal communities of different horizons from distinct tundra types; (**b**) RDA biplot of fungal diversity and environmental factors. Significant effect of soil parameters (black arrow in figure) on fungal communities were identified by forward selection. The proportion of variability explained by significant soil parameters are given in the lower right corner.

We performed a permutational multivariate analysis of variance (PERMANOVA) to determine the effect of different horizon and tundra sites, both had a significant effect on fungal community composition (Figure 2). We found a stronger site (F-Model = 6.9, R^2^ = 0.15, *p*-value = 0.001) effect on fungal beta diversity than the horizon effect (F-Model = 1.9, R^2^ = 0.05, *p*-value < 1 × 10^−4^). Topsoil samples were clustered close to each other from Site 1 and Site 2, whereas two dispersed clusters were found for topsoil from Site 3. Samples from Site 4 were separated from other sites’ samples. The RDA-based forward selection was used to identify the most important environmental factors affecting the fungal communities, we found pH and DN as the main contributors.

### 3.3. Key Topological Properties of Co-Occurrence Network

**Figure 3:**
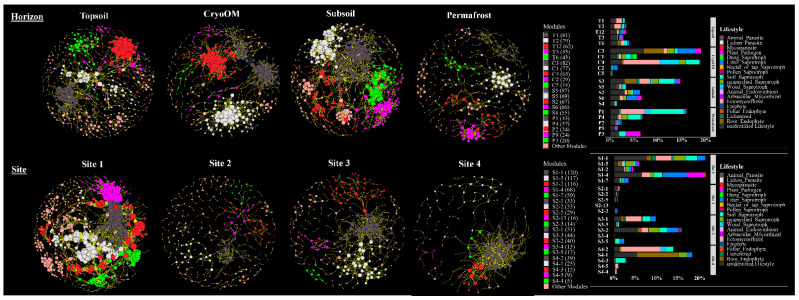
Co-occurrence network interaction of fungal zOTUs found in each horizon and tundra site. A connection stands for a strong Spearman’s correlation (*r* ≥ 0.6 and *p*-value ≤ 0.01). Each circle or node represented a fungal zOTU and the sizes of the circles were proportional to the values of node square-root degree. Lines connecting two fungal zOTU represented the interactions between them, yellow and blue lines represented the positive and negative significant correlations, respectively. Nodes were colored according to the top five modules. The relative proportion of fungal lifestyle from the top five modules of each horizon and tundra site are shown.

To identify the interaction of fungal taxa, we constructed a co-occurrence network from each horizon and tundra site (Figure 3). The respective global topological properties of the co-occurrence network with the corresponding random network are given in Table 2.

The number of nodes (zOTUs) that were significantly correlated was the highest in the topsoil samples (476), however, the number of significant correlations between zOTUs was greatest in the subsoil samples (3426). The number of total zOTUs after the abundance filtration was highest from Site 1 and lowest from Site 4. The higher number of zOTUs from Site 1 also accounted for a more connected co-occurrence network. We found Site 2 had a greater number of zOTUs compared to Site 4 but a considerably smaller number of significant correlations between zOTUs.

Co-occurrence network complexity is generally measured by DD and CC indexes. We found considerably different DD and CC indexes from the individual horizons. The higher the DD value, the more complex the network. Hence, the DD value suggested that the ecological network became more complex from the topsoil to the subsoil. The CC was highest in the cryoOM network compared to other horizons which suggested that the node’s neighbors were also connected in the cryoOM network. The DD values implied that the co-occurrence network from Site 1 was more complex, whereas the CC value indicated that the Site 4 network was more connected.

All generated networks were modular, as suggested by their modularity values which were higher than the suggested threshold value of 0.4 for modular structure [37] and higher than the corresponding random network (Table 2). A total of 31, 21, and 12 modules were obtained for topsoil, cryoOM, and subsoil, respectively, and 21, 40, 27, and 18 modules for Site 1, Site 2, Site 3, and Site 4, respectively. The relative proportion of the top five modules for each horizon and tundra site network at the trophic mode’s level is given in Figure 3. The top two modules (T1 and T2) from topsoil had a higher relative proportion of ectomycorrhizal, whereas C3 and C1 modules from the cryoOM co-occurrence network had a great relative proportion of litter saprotrophs and root endophytes, and dung and soil saprotrophs, respectively. The biggest module (97 nodes) in all horizons, S3, had a high relative proportion of soil saprotrophs, ectomycorrhizal, and root endophytes. In comparison to the horizons, the modules from the tundra site co-occurrence network were relatively smaller, except for Site 1 which had bigger modules. The biggest modules from individual tundra sites (S1-1, S3-1, and S4-2) had a greater relative proportion of ectomycorrhizal. Overall, the network structure was dramatically different between each horizon and tundra site, and also the shared nodes between them.

The shared nodes (zOTUs, identified from the co-occurrence network only) between horizons were lower compared to the unique nodes for the individual horizon networks (Figure S3). Whereas there were only four nodes shared between the individual tundra sites, Site 1 had the highest number of unique nodes (311).

**Figure 4:**
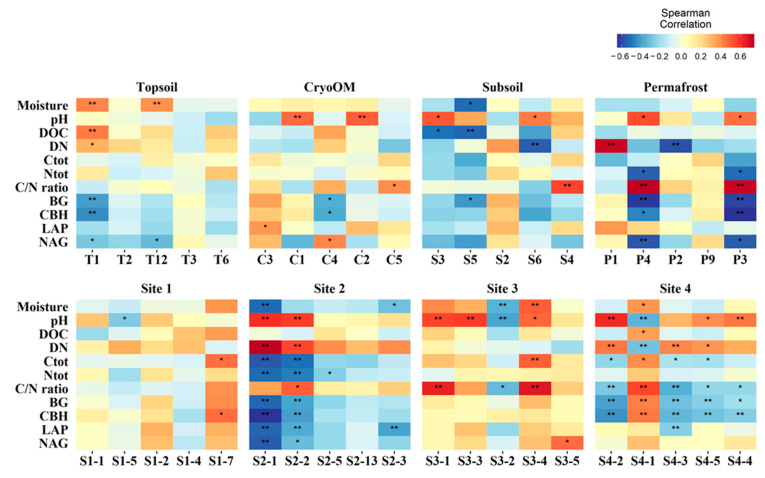
Spearman rank correlation coefficients of soil environmental factors and network modules (top five) for individual horizons and tundra sites. The reds represented a positive correlation and the blue represented a negative correlation. The heatmap cells marked by “*” or “**” were statistically significant: * *p*-value < 0.05 and ** *p*-value < 0.01.

We observed significant correlations between the network modules (top five only) and environmental variables (Figure 4). In topsoil modules, modules T1 and T12 which had a high relative proportion of ectomycorrhizal and litter saprotrophs, respectively, had a strong positive correlation with moisture and a significant negative correlation with NAG. Other than moisture, dissolved nutrients (DOC and DN) had a positive correlation and C enzyme (BG and CBH) activity had a negative correlation with topsoil module T1, whereas modules C1 and C2 from cryoOM had a strong positive correlation with pH. Ectomycorrhizal, which had a high relative proportion in module C4, was positively affected by NAG and negatively affected by BG and CBH. In the subsoil, soil saprotrophs, ectomycorrhizal, and root endophytes had a great relative proportion in module S3, and a significant positive correlation with pH and negative with DOC. The total number of significant correlations was highest in permafrost. A strong significant positive correlation was found between ericoid mycorrhizal comprised module P4 and both moisture and C/N ratio, whereas a negative correlation was observed with Ntot, BG, CBH, and NAG.

We found only three significant correlations between the network’s module and environmental factors from Site 1. These correlations included modules S1-5 and S1-7, which had a great relative proportion of unspecified saprotrophs and litter saprotrophs, negatively correlated with pH and positively correlated with Ctot and CBH, respectively. Modules S2-1 and S2-2 from Site 2 had a significantly negative correlation with Ctot, Ntot, BG, CBH, LAP, and NAG and a significantly positive correlation with pH and DN. We found a significant positive correlation between ectomycorrhizal comprised module S3-1 and pH and C/N ratio. Module S4-2, S4-3, S4-5, and S4-4 from Site 4 had a significant negative correlation with C/N ratio, BG, and CBH and the same environmental factor had a positive correlation with module S4-1 which had a great relative proportion of root endophytes.

### 3.4. The Topological Roles of Nodes and Generalist-Specialist Shift

**Figure 5:**
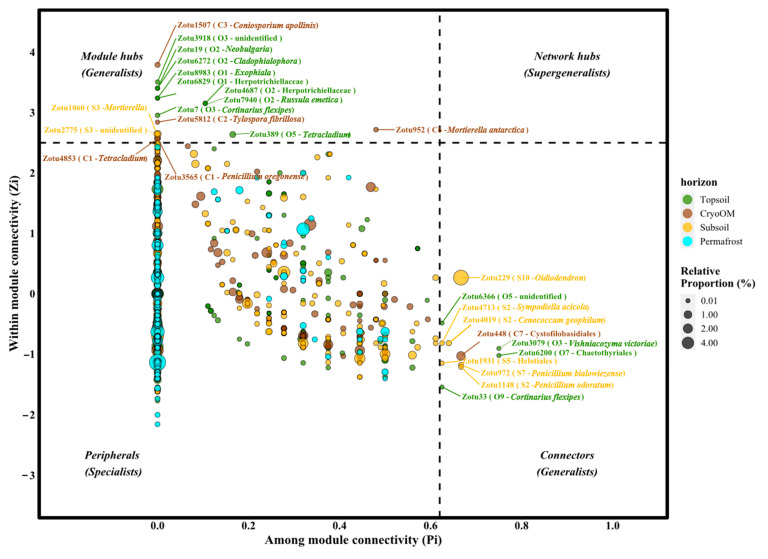

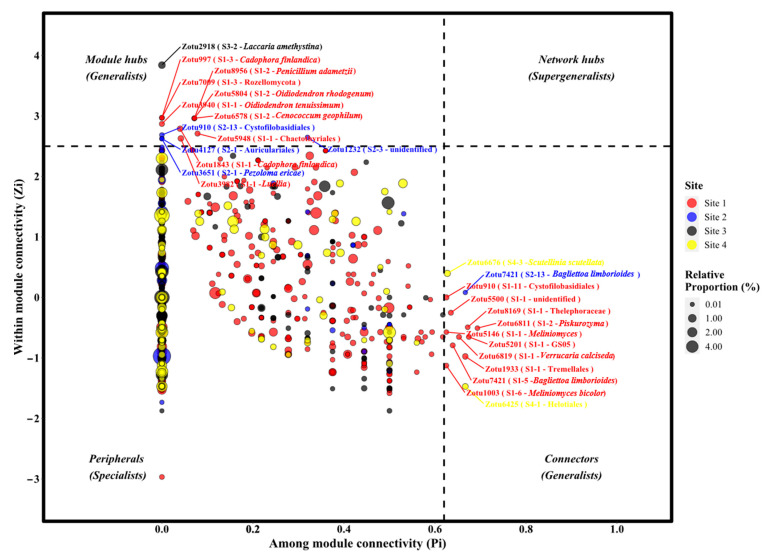
Zi-Pi plot showing topological roles of nodes in different horizons and tundra sites. The threshold values of Zi and Pi for categorizing nodes were 2.5 and 0.62, respectively. Generalists (connectors and module hubs) were labeled with zOTU IDs, module number, and maximum assigned taxonomy (bolded letters). Generalists were colored according to horizons and tundra sites and the size of each node represented the abundance of that node.

The topological roles of the nodes in networks were identified from the Zi-Pi plot (Figure 5), by plotting the within-module connectivity (Zi) and among-module connectivity (Pi) proposed by [74] and simplified by [70]. All nodes fell into four categories (peripherals, module hubs, network hubs, and connectors). We found that most nodes (97.2%, 98.2%, 98.1%, 100% for topsoil, cryoOM, subsoil, and permafrost, respectively) were peripherals that had a connection to other nodes but only in their own modules. Among them, 77.3% (topsoil), 76.1% (cryoOM), 65.4% (subsoil), and 57.3% (permafrost) of the peripherals had no edge outside of their own module (i.e., Pi = 0). In total, we found 11 nodes as connectors and 17 nodes as module hubs. A total of 4, 1, and 6 connectors and 9, 5, and 2 module hubs were found for topsoil, cryoOM, and subsoil networks, respectively. We did not find supergeneralists in any of the horizon’ networks. Detailed taxonomic information for the topological role is given in Table S4.

Similar to the horizon’s Zi-Pi plot, the Zi-Pi plot from the tundra site identified most of the nodes as a peripheral and a great number of those nodes (57.5%, 92.5%, 75.2%, and 68.4% for Site 1, Site 2, Site 3, and Site 4, respectively) did not have an edge outside their own module (Figure 5). Site 1 had the highest, and Site 3 had the lowest number of generalists (module hubs and connectors). In total, we found 10 connectors and 9 module hubs from Site 1.

It is also worth mentioning that some nodes were identified as generalists in one horizon but played the role of specialist (peripheral) in other horizons (Table 3 and Table S5). For instance, in topsoil, generalists included zOTU6200 (unidentified Chaetothyriales), zOTU4687 (unidentified Herpotrichiellaceae), and zOTU6829 (unidentified Herpotrichiellaceae), however, these zOTUs were found as specialists in other horizons. Similarly, generalists from cryoOM included zOTU952 (*Mortierella antarctica*), zOTU3565 (*Penicillium oregonense*), and zOTU4853 (unidentified *Tetracladium*), but these were observed as specialists in other horizons. Subsoil generalists included zOTU1148 (*Penicillium odoratum*) and zOTU2775 (unidentified Fungi), while these zOTUs were identified as specialists in other horizons. Similar to the horizon, we also found some zOTUs that identified as a generalist for one site but specialist for other sites, for instance, zOTU1843 (*Cadophora finlandica*) was identified as a generalist from Site 1 (module hubs) but specialist from all other sites (Table 4 and Table S5).

The fungal zOTU role shift may be attributed to different environmental factors. We found distinct correlation patterns between generalists and specialists with environmental factors from different horizons (Table S6) and tundra sites (Table S7). The distinct correlations, which to a certain extent indicated that the dominant factors shaping fungal networks were specific to each horizon and tundra site, and potentially change or shift the role of generalist−specialist (topological role shift).

In general, the fungal ecological networks in the topsoil, subsoil, and Site 1 contained more keystone taxa (generalists) than those in other horizons (cryoOM and permafrost) and sites (Site 2, Site 3, and Site 4), which may lead to a more effective organization of taxa connections in the network as they are regulated by more connectors and module hubs.

## 4. Discussion

Several studies have reported that high fungal diversity has a positive effect on ecosystem functioning, and a loss of fungal diversity can alter the ecosystem functioning, with changes such as lower enzyme activities and litter decomposition rates [75,76]. Additionally, soil fungi have specific substrate preferences and acquisition strategies. Hence, each of the soil fungi comprises different lifestyles and functions [77], and ultimately they form complex interactions with each other (i.e., competition, mutualism, predation, parasitism). These complex interactions determine the overall fungal community structures and stability [27,70,78]. In this study, we constructed a fungal co-occurrence network of different horizons and tundra sites based on high-throughput sequencing data of the fungal ITS region. Previous studies have reported differences between the fungal community structure of organic and mineral soils, for instance, a study from the high Arctic found a more diverse fungal community from the organic horizon than the mineral subsoil [8,79]. Only one previous study has focused on the fungal community composition from cryoOM soil [11]. Studies on the co-occurrence of microbial networks, on the other hand, provide essential information regarding the interaction between species in complex soil ecosystems. In this study, we constructed an ecological network of fine-scale taxonomy and identified important fungal interactions in the PAS.

### 4.1. Horizon and Tundra Specific Fungal Lifestyle

Differences in the soil fungal community across distinct horizons and tundra vegetation were apparent at the genera and lifestyle level, which suggest significant changes occur in the entire fungal community with depth and supports the theory that at least some degree of ecological coherence exists among different fungal lifestyles [80].

Our study showed that symbiotrophs are the most abundant functional lifestyle in PAS which is in agreement with studies from other ecosystems [22,81,82]. We also found that root endophytes had a greater relative proportion in topsoil compared to other horizons, however, not significant (Figure 1 and Figure S4). Root endophytes are plant-associated fungi that reside within plant tissues or grow inside roots, stems, or leaves, and they have been previously studied and isolated from Arctic vascular plants [83,84,85]. They have been shown to play an important role in the nutrient cycle of the other natural ecosystem, including the decomposition of Norway spruce needles [86,87]. Some species of endophytes exhibit functions morphologically and phylogenetically similar to saprotrophs and produce leaf degrading enzymes [88]. We, for instance, found the dark septate endophytic genus *Phialocephala* to have a significantly greater mean proportion in topsoil compared to other horizons (Figure 1 and Figure S2). Members of this genus utilize proteins as a sole nitrogen source [89], mineralize organic nitrogen in the rhizosphere [90], and potentially decompose SOM [91]. On the other hand, their relatively significant presence in the deeper layers of the PAS shows that endophytic fungi are not strictly tied to life inside plant tissues, but instead can migrate over relatively long distances in the soil, where they can participate in the decomposition of complex organic matter.

Recent studies show that other symbiotrophs such as mycorrhizal fungi can be viable competitors for saprotrophic fungi [92,93], but only under certain conditions. Due to their symbiotic plant friends, they gain a greater competitive advantage under C-limiting conditions in which the plant “pumps” its own C to them, which is used in part by the mycorrhizal fungus for the synthesis of extracellular enzymes [94,95]. These will help it “win” over the saprotrophic fungus. In this fight, mycorrhizal fungi have a competitive advantage where roots are present, but in deeper soil where roots are absent and mostly recalcitrant SOM dominates, it can be inhabited by litter saprotrophs. This seemingly minor battle can have a major impact on soil organic matter transformation in PAS. In our study, however, we found a significantly greater proportion of mycorrhizas in deeper soil of PAS in comparison to upper topsoil (Figure 1 and Figure S2). This can be explained either by the fact that plants root deeper on Herschel Island, which has not been confirmed, or that fungi in temperate ecosystems, known as mycorrhizal fungi, have multiple life strategies in the Arctic and can survive without a host in deeper horizons and feed saprotrophically. The litter saprotrophs are more efficient than mycorrhizal in colonizing and utilizing fresh, energy-rich compounds [96,97]. However, as the C/N ratio and available energy decrease with soil depth [98,99], saprotrophs might become less competitive, and be replaced by mycorrhizal fungi that do not depend on litter-derived energy in deep soil horizons [100]. We hypothesize that the energy and nutrient-demanding extracellular enzymes synthesized by mycorrhizal fungi utilize nutrient-rich compounds (mainly organic N), but because a large part of this N is then transported to plant symbiont, they remove N from the soil and leave C-rich and nutrient-poor substrates behind. Therefore, it may result in inadequate nutrient availability for the saprotrophic fungi in deeper soil, reducing SOM decomposition and potentially increasing C in PAS soil [101,102]. A previous study also reported that the C in cryoOM was thousands of years old and the decomposition process rate was slower and was three times older than the C in topsoil horizons [103]. CryoOM in soils is considered highly N-limited [99,104] and the greater proportion of ectomycorrhizal fungi which are known to have a less efficient enzyme activity [100,105] compared to saprotrophs can exacerbate this limitation and potentially increase C storage in PAS. We argue that the “role shift” of mycorrhizal lifestyle in topsoil to a more saprotrophic lifestyle in deeper soil horizons can affect the vulnerability of C in PAS.

### 4.2. Co-Occurrence Networks Reveal More Complex Interactions in Deeper Soil Horizons

The analysis of co-occurrence patterns can provide a vivid and simplified version of the interactions in complex fungal communities. Moreover, it offers an in-depth insight into ecological assembly from different horizons and sites.

We found that fungal assemblages in topsoil formed a less complex network compared to those in the subsoil, even with the highest number of nodes and significantly higher fungal gene abundance among all horizons. Topsoil in the Arctic is experiencing extreme changes (i.e., a higher fluctuation temperature and nutrient cycling) compared to deeper soil horizons. This may have forced selective pressure on the fungal communities, which was also evidenced by the high fungal richness but unevenly distributed fungal communities (Table S3). This was reflected in a less connected network in topsoil compared to the subsoil. A relationship between species richness, diversity, and network connectivity has been previously observed [30,106]. It was suggested that microbial diversity decreases as network size and connectivity increase. Furthermore, an increased network complexity with increased soil depth (in subsoil) for bacterial and fungi were previously observed in a grassland study [107]. We hypothesize that the more densely connected network of fungal communities in deeper soil horizons is due to the oligotrophic environment of these horizons, where different groups of fungi must compete or cooperate to obtain nutrients that are in short supply. This may be due to the decreased direct input of root exudates and possible metabolic recalcitrant byproducts that remain in the lower soil horizons. These conditions could generate more competition or co-metabolism due to the lower quality and quantity of substrate available in deeper soil horizons. In support of this idea, negative correlation, which suggests co-exclusion between two taxa, increased from the topsoil to the subsoil network (Table 2). This trend may indicate a more competitive (negative correlation) relationship between fungal species in deeper soil horizons compared to topsoil [108]. Moreover, APL and CC were lowest and highest, respectively in cryoOM compared to all other horizons. Networks that have smaller APL and higher CC are considered a “small world” which means every species is connected to every other species through a very short path and an “all-my-friends-know-each-other” relationship [109,110]. The networks termed “small worlds” are generally vulnerable to the rapid changes of an ecosystem perturbation [111]. Therefore, fungal communities from the cryoOM may be more sensitive to environmental changes compared to other horizons. It may also reflect a less fluctuating environment compared to that experienced by topsoil.

We found a great difference in the co-occurrence network for different tundra sites too, Site 1 was more complex, whereas nodes from Site 4 were more connected. The potential reason was that the fungi had a higher richness and Shannon index from Site 1 compared to other sites, thus causing more complex fungal interaction [30,106]. Whereas Site 4 had the lowest richness and diversity which made it a less complex but more connected co-occurrence network as suggested above for the topsoil horizon. Moreover, APL and CC values suggested that Site 4 is a “small world” and vulnerable to environmental changes.

### 4.3. Greater Connectivity but Lower Specialization

The modules in ecological networks play a critical role in maintaining overall microbial community structure and stability, hence, the majority of ecosystem studies have focused on identifying modules in ecological networks [27,70,112,113]. Modules, by definition, are densely connected nodes that have more edges inside the module than outside. From our study, we found an average modularity higher than 0.4 which suggested a modular structure in all horizons and tundra sites [37].

The modularity value was lowest in buried cryoOM and Site 4 and highest in the mineral subsoil and Site 2 (Table 2). This highly modular network means that the fungal community is stable with an ordered structure with high efficiency at nutrient and information exchange [68]. Previous studies have interpreted modules as niches [114,115], and we found higher modularity values within subsoil and Site 2 which linked to stronger niche separation compared to other horizons and tundra sites.

### 4.4. Environmental Condition Associated with Topological Role Shift

In the present study, connectors and module hubs were considered as generalists and peripherals (taxa in the network which have only a few connections and only within their own module) as specialists [27]. Generalists are the key fungi that promote the exchange of nutrients and information among different taxa in network and hence play a pivotal role in maintaining the balance between different microbial taxa. In a natural ecosystem, generalists uptake nutrients from a broad range of sources and grow well in many habitats, whereas specialists have very specific nutrient requirements and therefore their growth is restricted to some habitats only [27,45,112,116]. In total, we found 13, 6, and 8 generalists within the topsoil, cryoOM, and subsoils, respectively. Additionally, the role of some taxa shifted in different horizons, topsoil (zOTU6200, unclassified Chaetothyriales; zOTU4687, *Cladophialophora*; and zOTU6829, *Cladophialophora*), cryoOM (zOTU952, *Mortierella antarctica*; zOTU3565, *Penicillium oregonense*; and zOTU4853, *Tetracladium*), and subsoil (zOTU1148, *Penicillium odoratum* and zOTU2775, unidentified fungi) were found as generalists in the respective horizons but a specialist in other horizons (Table 3). The role shifts of a generalist to a specialist in cryoOM probably occurred as a result of major events whereby the topsoil community was buried into deep soil horizons and surrounded by mineral subsoil with low nutrient availability and higher competition pressure between taxa. Two lines of evidence supported this generalization. Firstly, the generalist taxa in the topsoil network were found to be specialist taxa in the cryoOM network, suggesting their role shift (Figure 5 and Table S5). Secondly, the majority of taxa identified in the cryoOM network were not shared with topsoil network taxa (Figure S3), but with subsoil network taxa [11,117].

The number of generalists identified from each tundra site was 19, 5, 1, and 2 from Site 1, Site 2, Site 3, and Site 4, respectively. The taxa which were identified as generalists from Site 1 but specialists from other sites were litter saprotrophic zOTU1843 (*Cadophora finlandica*) (Table 4). This taxon has shown the ability to degrade various polysaccharides including cellulose, starch, and xylem [89], and previously detected from the Canadian High Arctic [118]. We speculate that the reason for this taxon being a generalist from Site 1 was mainly because Site 1 was mostly dominated by vascular plant vegetation (cotton grass) which has more above-ground biomass compared to the other sites’ vegetation. Tussock cotton grass generally has more dead leaves and culms than the living which may also nourish the higher proportion of litter-decomposing saprotrophic fungi. The role shift of key fungal taxa can be attributed to the different environmental conditions experienced in different horizons (Table S6) and tundra sites (Table S7).

The generalist taxa from topsoil but specialists from other horizons belonged to the order Chaetothyriales. Fungi from this order are dark septate root endophytes and as described above, they commonly interact with plant roots which may explain their role as generalists in topsoil. In contrast, in deeper soil horizons where plant roots are less abundant and also the availability of nutrients is scarcer, they might have different roles to play. For example, members of *Cladophialophora* were found as mycoparasites [119,120], and due to the lower nutrient availability in deeper soil, they might feed on other fungal species. This is in support of the fact that overall fungal gene abundance was found to be lower in cryoOM and subsoil compared to upper topsoil from this study and a previous study [11]. The generalists from cryoOM, had a negative correlation with LAP activity from topsoil but no such correlations were observed from cryoOM, we found a positive correlation with NAG activity instead. We speculated that high LAP production by other taxa (i.e., *Articulospora*) might have a negative effect on these taxa and potentially change their role to specialists. Furthermore, significantly higher DN content in subsoil and permafrost compared to the other two horizons (Table 1), potentially contributed to shifting the role of generalist from cryoOM to specialist in subsoil and permafrost.

Collectively, these co-occurrence data suggest that role shifts probably happen when the top layer becomes buried in the deep soil layer, and more connectors being shared between cryoOM and subsoil may suggest that most of these changes in cryoOM were driven by the different environmental conditions in surrounding mineral subsoil and the resident fungi [11,117].

## 5. Conclusions

In conclusion, our data showed that different horizons and tundra sites of the active layer of cryosols harbored not only distinct fungal communities with diverse lifestyles but also specific co-occurrence patterns along with changes in topological role (from generalist to specialist and vice-versa). The interactions of distinct microbial taxa can be more important to soil processes than species richness and their abundance, more importantly in the ecosystem where extreme changes happen in a short time. The inference of microbial networks allows us to find key microbes which are pivotal in maintaining the overall community structure and perform key roles.

Ultimately, such co-occurrence network analysis will be able to predict the outcome of community alterations (topological role shift) and the effects of environmental perturbations. For example, members of *Cladophialophora* were found as generalists in upper PAS where microbes are not limited by nutrients, but in the deeper soil layer, where nutrients are scarcer, they shifted their role from being a generalist to a specialist (mycoparasite) due to nutrient constraints. The topological indexes, average path length, and clustering coefficient suggested that the fungal network from cryoOM is a small world where everyone is connected to each other with a short path and every taxon is known to each other. The small world (cryoOM) is suggested to be more vulnerable to environmental changes than the bigger world (topsoil), thus, perturbation may lead to change in the overall carbon storage in PAS. The taxon *Cadophora finlandica* (litter saprotrophs) was identified as a generalist from Site 1 where litter is in ample amounts, however, for the other site its role shifted to a specialist due to environmental constraints.

Although exploring such an ecological network improves our understanding of microbial ecology, more investigations are needed to overcome methodological limitations such as the prediction of a relationship between two taxa by interpreting the correlation. For instance, the incorporation of techniques that will not only take into account the relationship between two taxa but also third-party microorganisms and random soil processes. In addition, limited information on biotic and abiotic factors that covary in different horizons demands further investigation to determine the exact drivers and mechanisms of topological role shifts (generalist to a specialist), the number of which increased from topsoil to permafrost.

## Figures and Tables

**Table 1:** Soil environmental factors in each horizon. Averages and standard deviation were shown. The significant difference between different horizons within all tundra sites were calculated using One-Way ANOVA and followed by a Tukey’s HSD test. Different letters in the brackets indicated a significant difference between tundra sites.

Site	Horizon	N	Moisture (%)	pH	DOC (ug/g dw)	DN (ug/g dw)	Ctot (%)	Ntot (%)	C/N ratio	BG (nmol MUF g^−1^ dw h^−1^)	CBH (nmol MUF g^−1^ dw h^−1^)	LAP (nmol MUF g^−1^ dw h^−1^)	NAG (nmol MUF g^−1^ dw h^−1^)
Site 1	Topsoil	9	76.6 ± 3.45 (a)	6 ± 0.22 (a)	751.08 ± 308.98 (a)	2.56 ± 0.9 (b)	40.06 ± 3.43 (a)	1.15 ± 0.22 (a)	44.76 ± 14.8 (a)	1624.9 ± 294.78 (a)	239.86 ± 76.91 (a)	186.85 ± 79.3 (a)	400.23 ± 83.42 (a)
	CryoOM	7	50.89 ± 6.91 (b)	6.35 ± 0.19 (a)	382.47 ± 84.75 (a)	8.3 ± 3.38 (ab)	11.87 ± 3.95 (b)	0.77 ± 0.24 (ab)	15.11 ± 0.57 (b)	441.33 ± 375 (b)	101.23 ± 107.23 (a)	74.09 ± 66.4 (a)	144.61 ± 40.54 (b)
	Subsoil	5	30.44 ± 3.31 (c)	5.81 ± 0.05 (a)	205.97 ± 63.69 (a)	9.92 ± 1.22 (a)	4.63 ± 1.11 (b)	0.33 ± 0.07 (b)	13.48 ± 0.64 (b)	75.32 ± 26.19 (b)	13.47 ± 3.88 (a)	15.39 ± 3.48 (a)	76.08 ± 22.59 (b)
	Permafrost	2	55.2 ± 5.89 (ab)	5.93 ± 0.18 (a)	695.72 ± 187.2 (a)	7.68 ± 1.56 (ab)	8.2 ± 0.33 (b)	0.56 ± 0.03 (ab)	14.78 ± 0.25 (b)	115.51 ± 7.6 (b)	13.16 ± 3.89 (a)	10.67 ± 4.41 (a)	73.9 ± 10.39 (b)
Site 2	Topsoil	8	54.51 ± 4.08 (a)	7.49 ± 0.29 (b)	466.72 ± 142.64 (a)	3.19 ± 1.46 (b)	29.57 ± 4.66 (a)	1.27 ± 0.13 (a)	22.93 ± 2.25 (b)	1595.35 ± 381.81 (a)	268.63 ± 72.32 (a)	704.2 ± 140.03 (a)	389.05 ± 174.6 (a)
	CryoOM	2	54.26 ± 2.53 (a)	8.05 ± 0.11 (ab)	250.78 ± 11.86 (a)	2.64 ± 0.48 (b)	16.13 ± 1.29 (b)	1.03 ± 0.03 (a)	15.6 ± 0.75 (b)	263.6 ± 96.89 (b)	36.63 ± 18.79 (b)	128.23 ± 24.4 (b)	109.24 ± 2.79 (ab)
	Subsoil	7	23.86 ± 3.87 (b)	8.12 ± 0.23 (ab)	326.18 ± 65.47 (a)	19.58 ± 6.76 (ab)	4.15 ± 0.71 (c)	0.22 ± 0.06 (b)	21.22 ± 2.2 (b)	21.58 ± 17.59 (b)	1.56 ± 1.33 (b)	77.66 ± 45.09 (b)	6.66 ± 5.14 (b)
	Permafrost	9	29.04 ± 4.5 (b)	8.24 ± 0.3 (a)	374.77 ± 134.22 (a)	31.19 ± 11.31 (a)	3 ± 0.13 (c)	0.1 ± 0.01 (b)	29.52 ± 1.76 (a)	1.02 ± 0.47 (b)	0.15 ± 0.12 (b)	25.35 ± 4.21 (b)	0.58 ± 0.37 (b)
Site 3	Topsoil	21	78.32 ± 4.17 (a)	5.94 ± 0.38 (a)	5104.09 ± 2839.27 (a)	14.5 ± 7.45 (a)	30.2 ± 4.74 (a)	1.74 ± 0.25 (a)	17.93 ± 3.19 (a)	690.13 ± 399.39 (a)	90.1 ± 75.82 (a)	94.67 ± 73.1 (a)	241.01 ± 116.29 (a)
	CryoOM	5	54.11 ± 9.86 (b)	6.11 ± 0.39 (a)	1066.41 ± 719.77 (a)	22.09 ± 9.95 (a)	14.57 ± 3.26 (b)	1.06 ± 0.2 (b)	13.72 ± 1.6 (a)	175.59 ± 108.25 (a)	20.53 ± 13.51 (a)	16.18 ± 4.58 (a)	119.29 ± 31.8 (a)
	Permafrost	1	60.4	5.61	451.92	6.87	14.64	1.22	11.97	206.35	25	12.02	46
Site 4	Topsoil	12	75.57 ± 6.73 (a)	5.35 ± 0.42 (b)	2023.25 ± 628.9 (a)	6.86 ± 3.88 (c)	37.97 ± 6.07 (a)	1 ± 0.26 (a)	44.73 ± 12.06 (a)	1336.83 ± 371.99 (a)	204.45 ± 77.38 (a)	59.27 ± 40.06 (a)	271.86 ± 110.15 (a)
	CryoOM	16	59.85 ± 9.11 (a)	5.93 ± 0.23 (b)	1588.26 ± 1028.78 (a)	13.9 ± 4.18 (bc)	17.44 ± 3.81 (b)	0.87 ± 0.12 (a)	19.68 ± 2.59 (b)	473.34 ± 422.09 (b)	110.9 ± 134.8 (a)	53.8 ± 25.98 (a)	316.64 ± 104.08 (a)
	Subsoil	10	29.9 ± 6.03 (b)	5.91 ± 0.37 (b)	442.22 ± 138.73 (a)	38.54 ± 10.32 (b)	4.23 ± 0.98 (c)	0.22 ± 0.04 (b)	18.65 ± 2.12 (b)	99.28 ± 55.15 (b)	11.69 ± 4.79 (a)	35.73 ± 9.39 (a)	37.7 ± 23.6 (b)
	Permafrost	8	38.1 ± 11.77 (b)	7.32 ± 0.56 (a)	671.93 ± 477.72 (a)	100.06 ± 24.95 (a)	3.17 ± 0.64 (c)	0.21 ± 0.04 (b)	15.22 ± 0.88 (b)	60.28 ± 52.38 (b)	7.04 ± 7.78 (a)	27.92 ± 9.69 (a)	36.17 ± 33.42 (b)

**Table 2:** Major topological properties of the empirical networks of soil fungal communities in different horizons and tundra sites, and their associated random network.

	Horizon	Topsoil	CryoOM	Subsoil	Permafrost	Site 1	Site 2	Site 3	Site 4
Empirical network	Total zOTUs ^a^	558	413	479	479	688	458	324	212
	Abundance (%) ^b^	61.13	68.22	63.22	71.06	75.97	67.33	65	71.36
	Total significant correlations	3054	2454	3426	1110	6886	666	1152	814
	Total node	476	340	437	281	643	267	246	130
	Total edge	1527	1227	1713	555	3443	333	576	407
	Positive edge	1527	1216	1699	553	3277	331	568	407
	Negative edge	0	11	14	2	166	2	8	0
	Average path length (APL)	7.12	5.3	5.42	7.61	5.72	7.2	5.4	5.23
	Degree distribution (DD)	6.42 ± 7.88	7.22 ± 7.05	7.84 ± 6.05	3.95 ± 3.3	10.71 ± 9.83	2.49 ± 1.89	4.68 ± 4.66	6.26 ± 7.64
	Average closeness (AC)	−4.42 ± 0.28	−4.16 ± 0.28	−3.82 ± 0.21	−4.5 ± 0.24	−4.33 ± 0.25	−4.67 ± 0.16	−4.23 ± 0.27	−3.82 ± 0.23
	Average betweenness (AB)	1225.6 ± 2195.08	606.04 ± 1405.7	923.8 ± 1432.28	494.75 ± 1148.79	1404.42 ± 1920.9	246.18 ± 597.43	353.79 ± 728.76	153.46 ± 332.88
	Edge density (ED)	0.0135	0.0213	0.018	0.0141	0.0167	0.0094	0.0191	0.0485
	Diameter (D)	20	16	15	20	17	19	17	17
	Clustering coefficient (CC)	0.24	0.34	0.23	0.26	0.18	0.13	0.23	0.3
	Number of modules	31	21	12	34	21	40	27	18
	Modularity (M)	0.72	0.68	0.73	0.82	0.69	0.85	0.65	0.38
Random network ^c^	Average path length (APL)	3.52 ± 0.008	3.16 ± 0.007	3.18 ± 0.005	4.2 ± 0.042	2.44 ± 0.001	3.64 ± 0.022	2.7 ± 0.005	2.17 ± 0.005
	Clustering coefficient (CC)	0.01 ± 0.002	0.02 ± 0.003	0.02 ± 0.002	0.01 ± 0.004	0.03 ± 0.001	0.02 ± 0.004	0.04 ± 0.003	0.1 ± 0.005
	Modularity (M)	0.32 ± 0.01	0.3 ± 0.011	0.28 ± 0.01	0.44 ± 0.016	0.15 ± 0.004	0.38 ± 0.014	0.26 ± 0.011	0.21 ± 0.012

^a^ total zOTUs left after filtration; ^b^ total abundance of filtrated zOTUs; ^c^ same number of nodes and edges from empirical networks were used to calculated random network.

**Table 3:** Topological role shift between different horizons. Module hubs and connectors were considered as generalists whereas peripheral as specialists.

zOTUs ID	Genera	Species	Lifestyle	Topsoil	CryoOM	Subsoil	Permafrost
Zotu6272	*Cladophialophora*	*unidentified*	Soil_Saprotroph	Module hubs	Peripheral	Peripheral	-
Zotu7	*Cortinarius*	*Cortinarius flexipes*	Ectomycorrhizal	Module hubs	-	-	Peripheral
Zotu952	*Mortierella*	*Mortierella antarctica*	Soil_Saprotroph	Peripheral	Module hubs	Peripheral	Peripheral
Zotu1060	*Mortierella*	*unidentified*	Soil_Saprotroph	Peripheral	Peripheral	Module hubs	-
Zotu19	*Neobulgaria*	*unidentified*	Wood_Saprotroph	Module hubs	-	Peripheral	Peripheral
Zotu229	*Oidiodendron*	*unidentified*	Soil_Saprotroph	-	Peripheral	Connectors	Peripheral
Zotu972	*Penicillium*	*Penicillium bialowiezense*	unspecified_Saprotroph	Peripheral	-	Connectors	Peripheral
Zotu1148	*Penicillium*	*Penicillium odoratum*	unspecified_Saprotroph	Peripheral	Peripheral	Connectors	Peripheral
Zotu3565	*Penicillium*	*Penicillium oregonense*	unspecified_Saprotroph	Peripheral	Module hubs	Peripheral	Peripheral
Zotu7940	*Russula*	*Russula emetica*	Ectomycorrhizal	Module hubs	-	Peripheral	-
Zotu4713	*Sympodiella*	*Sympodiella acicola*	Litter_Saprotroph	-	-	Connectors	Peripheral
Zotu389	*Tetracladium*	*unidentified*	Litter_Saprotroph	Module hubs	-	-	Peripheral
Zotu4853	*Tetracladium*	*unidentified*	Litter_Saprotroph	Peripheral	Module hubs	Peripheral	Peripheral
Zotu5812	*Tylospora*	*Tylospora fibrillosa*	Ectomycorrhizal	-	Module hubs	Peripheral	-
Zotu3079	*Vishniacozyma*	*Vishniacozyma_victoriae*	Soil_Saprotroph	Connectors	-	-	Peripheral

**Table 4:** Topological role shift between different tundra sites. Module hubs and connectors were considered as generalists whereas peripheral as specialists.

zOTUs ID	Genera	Species	Lifestyle	Site 1	Site 2	Site 3	Site 4
Zotu1843	*Cadophora*	*Cadophora finlandica*	Litter_Saprotroph	Module hubs	Peripheral	Peripheral	Peripheral
Zotu6578	*Cenococcum*	*Cenococcum geophilum*	Ectomycorrhizal	Module hubs	Peripheral	-	-
Zotu3982	*Luellia*	*unidentified*	Wood_Saprotroph	Module hubs	Peripheral	Peripheral	-
Zotu5146	*Meliniomyces*	*unidentified*	Root_Endophyte	Connectors	Peripheral	Peripheral	-
Zotu6819	*Verrucaria*	*Verrucaria calciseda*	Lichenized	Connectors	Peripheral	-	-

## Data Availability

The raw sequence generated for this study can be found in the European Nucleotide Archive (ENA) under the study of PRJEB44296.

## Supplementary Materials

The following are available online at https://www.mdpi.com/article/10.3390/microorganisms9091943/s1, Figure S1: Significantly different in mean proportion of fungal genera for the different horizon and tundra sites. Only >1% mean proportion and significant difference between one horizon or site to rest of the horizons or sites are shown. Different colored bars represent topsoil, green; cryoOM, brown; subsoil, yellow; permafrost, cyan; Site 1, red; Site 2, blue; Site 3, black; Site 4, yellow; Figure S2: Significantly different in mean proportion of fungal lifestyle for different horizon and tundra site. Only >1% mean proportion and significant difference between one horizon or site to rest of the horizons or sites are shown. Different colored bars represent topsoil, green; cryoOM, brown; subsoil, yellow; permafrost, cyan; Site 1, red; Site 2, blue; Site 3, black; Site 4, yellow; Figure S3: Venn diagram of the total number of nodes (zOTUs) overlapping between different horizons and site networks and percentages of overlapping are given in the brackets; Table S1: Soil environmental factors in each tundra site. Averages and standard deviation are shown. The significant difference between tundra sites within all horizons together and individual horizons were calculated using one-way ANOVA and followed by a Tukey’s HSD test. Different letters in the brackets indicate a significant difference between tundra sites; Table S2: Fungal relative proportion at the genera level for individual samples; Table S3: Fungi α-diversity indices. Averages and standard deviation are shown. The significant difference between all horizons and site together and within site were calculated using one-way ANOVA and followed by a Tukey’s HSD test. Different letters in the brackets indicate a significant difference between horizons and sites; Table S4: Taxonomy of zOTUs identified by Zi-Pi plot; Table S5: zOTUs identified either module hub or connect (generalist) for one horizon or tundra site, but peripheral (specialist) for other horizons and tundra sites; Table S6: Correlation between keystone taxa (connectors and module hubs) identified from the Zi-Pi plot and environmental factors. Only significant correlations are shown. zOTUs ids were followed by letters in brackets which denote T, topsoil; C, cryoOM; S, subsoil; and P, permafrost. These letters mean that zOTU was identified as specialists from other horizons as well; Table S7: Correlation between keystone taxa (connectors and module hubs) identified from the Zi-Pi plot of tundra site and environmental factors. Only significant correlations are shown. zOTUs ids were followed by the number in brackets which denote 1, Site 1; 2, Site 2; 3, Site 3; and 4, Site 4. These numbers mean that zOTU was identified as specialists from other sites as well.
